# Intra-individual comparison of appetitive trace and delay conditioning in humans across acquisition and extinction

**DOI:** 10.1038/s41598-025-05350-0

**Published:** 2025-06-20

**Authors:** Johannes B. Finke, Anna M. Schippers, Tim Klucken

**Affiliations:** Department of Psychology, Clinical Psychology and Psychotherapy, University of Siegen, Siegen, Germany

**Keywords:** Appetitive conditioning, Trace conditioning, Extinction learning, Pupil dilation, Startle modulation, Heart-period modulation, Human behaviour, Neurophysiology, Classical conditioning, Reward

## Abstract

Temporal contiguity between conditioned (CS) and unconditioned stimuli (US) is a crucial factor in Pavlovian learning, yet little is known about its role in appetitive conditioning and extinction. In a within-subject design, 60 participants underwent both a delay (DC) and trace conditioning (TC) session with partial reinforcement (75%) by monetary rewards (US) and varying interval between CS offset and US onset (DC: 0s; TC: 4s). In addition to self-report indices (reward expectancy, arousal, valence), psychophysiological markers (pupil dilation, heart-period and startle reflex modulation) were recorded during acquisition and extinction training. For most measures, significant differential conditioned responses emerged, irrespective of temporal contiguity, with no major differences observed between TC and DC during acquisition (except for potentially diminished startle attenuation in TC). Despite overall similar patterns in conditioned responding (with small to moderate effects on physiological measures), there was no intraindividual concordance between sessions, yet evidence for differential TC effects on extinction learning. Specifically, smaller reductions in differential reward expectancy, heart-period deceleration and startle modulation after extinction in TC suggested relatively diminished extinction learning. Conditioned pupil dilation (0–2 s after CS onset) remained comparatively stable. Taken together, our findings extend evidence of differences in underlying learning mechanisms between TC and DC to the context of reward learning.

**Supplementary Information:**

The online version contains supplementary material available at 10.1038/s41598-025-05350-0.

## Introduction

Learning to predict future events based on environmental cues is critical to an organism’s survival. A famous model of such learning is Pavlovian conditioning^1^, which is assumed to be ubiquitous across species, contexts and modalities^2^. In many real-world examples, however, sensory cues and motivationally relevant outcomes are not always perceived as spatiotemporally overlapping or contiguous (e.g., the smell of smoke as an index of fire), but rather separated by considerable temporal ‘gaps’ (e.g., the distant, fleeting sound of a predator approaching, or the tracks of potential prey). Recognizing the underlying predictive relationship thus requires bridging these gaps by storing both a representation of the cue and its contingency with the expected outcome in memory (rather than simply linking two active sensory representations). Due to this lack of temporal contiguity, presumably necessitating to encode representations of time^3^, *trace conditioning* (TC) has been theorized to be computationally more complex than classical *delay conditioning* (DC)^4^ and has often been linked to awareness in humans^5^. While both types of Pavlovian conditioning involve a process in which the onset of an initially neutral, conditioned stimulus (CS) is followed by an appetitive or aversive unconditioned stimulus (US), in DC the US is presented either immediately after the CS or in parallel (with a brief overlap), while in TC an inter-stimulus interval (between CS offset and US onset) is inserted during which both stimuli are absent. To date, surprisingly few studies have directly compared TC and DC to identify differences and similarities. In humans, TC has mostly been investigated with respect to threat of shock (usually referred to as ‘fear conditioning’; e.g^6–8^), or other, mildly aversive stimulation such as tactile eyeblink responses elicited by air puffs; e.g^9–11^). Despite some overlap in neural substrates (such as the cerebellum^12^), this research suggests that TC and DC depend on partially distinct brain networks (with TC relying particularly on hippocampal and prefrontal activity^7,13,14^, whereas the cerebellum may play a relatively more important role in DC^15^). Consequently, both trace eyeblink^9,10^ and trace fear conditioning^6,16^ are thought to require conscious learning in contrast to DC (but see^17^). However, very little is known empirically about appetitive TC, despite its obvious relevance for understanding the neurocognitive processes that underlie craving and addiction^18^. Notably, some findings from rodent research suggest a dissociation between the brain systems implicated in aversive TC (especially the hippocampus) and the mechanisms underlying appetitive TC^19,20^ (but see^21^). While there is some human research using similar tasks that involve a gap between cue and reward administration, these experimental paradigms (e.g., the ‘Incentive Monetary Delay Task’^22^) often include an additional instrumental component (e.g., go-no go task), limiting their generalizability with respect to CS-US contingency learning. Most importantly, a systematic, experimental comparison of appetitive TC and DC in humans is missing. The present study aimed to fill this gap by comparing a simple DC paradigm with monetary rewards to TC in terms of conditioned responses (CRs) across a variety of self-report (reward expectancy, arousal, valence) as well as state-of-the-art psychophysiological indices (pupil dilation [PD], heart-period [HP] and acoustic startle reflex [ASR] modulation). While no single peripheral-physiological index is yet firmly established as a marker of appetitive conditioning in humans, these measures were selected based on their prevalent use in human fear conditioning research^23^ as well as promising findings from the literature on reward learning. (Note that skin-conductance responses [SCR] were initially also recorded, but we refrained from an in-depth analysis of SCR data due to the low proportion of valid responses; see Methods.) Both PD^24–28^ and HP responses^24,29,30^ have been suggested as sensitive markers for tracking appetitive differential conditioning across trials, and there is also evidence for attenuation of startle by appetitively conditioned cues^31,32^ (similar to^32^, but in contrast to some previous studies, we opted for measuring ASR modulation only after the initial acquisition phase to rule out that aversive stimulation might interfere with initial CR acquisition^33^). To facilitate a direct comparison between the two types of learning, all participants took part in both a DC and TC session (on separate days) with visual cues as CS. Minor (counterbalanced) changes in stimulus materials notwithstanding, the two sessions were virtually identical except for a 4-s trace interval inserted between CS and US presentation in TC (contrasted with a 0-s gap in DC). Despite being a secondary (i.e., learned) reinforcer, monetary rewards have some favorable features in the present context, particularly their feasibility regarding precise timing (necessary for contrasting TC and DC), their ubiquity (i.e., relatively low susceptibility to individual differences in preference) and comparability with previous human research (as money has frequently been used in both classic and instrumental conditioning tasks). Overall, a large body of research has demonstrated the effectiveness of money for inducing differential CRs both at the behavioral and neurophysiological level (e.g^27,34^), although primary reinforcers (such as food) may also provide distinct advantages in certain contexts (such as their higher translational value).

Based on findings from the fear conditioning literature (e.g^6–8,16^), we anticipated overall similar patterns of CRs during acquisition in TC and DC. However, some studies have suggested contingency awareness to play a greater role in TC^6,16^, which is generally considered to be more demanding of cognitive, especially attentional, resources^35^. Also, CRs in TC often appear to be somewhat less pronounced than in DC, even though this may not necessarily reflect weaker associative strength^4^. Therefore, the main question underlying the present comparison between TC and DC was whether the temporal gap between CS and US might disrupt contingency learning to some extent, leading to reduced and/or less stable acquisition of differential CRs in TC or, vice versa, more pronounced and consistent conditioning effects in DC. However, it is also conceivable that this difference between learning conditions emerges mainly in self-report measures (indicative of the cognitive component of learning), whereas some psychophysiological markers (startle modulation in particular^6^) may be more sensitive to implicit learning. To complicate matters further, it cannot be ruled out that the assumed engagement of additional brain systems in TC^7^ fully compensates for (and thus masks) this putative effect on overt response systems.

To examine not only the acquisition of differential CRs, but also potential effects of TC on extinction learning processes, a subsequent ‘extinction training’ phase was included as well, designed to induce a gradual decay of reward expectancy. Specifically, to enhance the sensitivity of the paradigm to variation in (partial) extinction of CRs as a function of prior learning, a partial reinforcement schedule (75%) was used during initial conditioning, known to slow down extinction learning^36^. Since contextual changes have also been found to impact heavily on extinction memory^37,38^, the acquisition phase was followed (after a short break) first by another brief conditioning phase with unchanged contingency (‘post-acquisition’) and then by immediate extinction training (without continued reinforcement). The rationale for including the post-acquisition phase prior to extinction training was two-fold: (1) to allow for the assessment of startle responses *after* acquisition, yet under conditions of unchanged reward contingencies; (2) to minimize contextual effects and to facilitate a partial (rather than rapid) extinction of CRs (by masking the onset of extinction training and reducing the salience of the temporal ‘break’). Notably, it has been hypothesized that, similar to well-known effects of intermittent in contrast to continuous reinforcement^39^, extinction of CRs may be slower in TC than in DC. Such an effect may result from the lack of direct stimulus-stimulus associations in TC, potentially increasing uncertainty about expected outcomes, but also from the (presumed) associated higher recruitment of attentional resources^35,40^, which may lead to deeper encoding and/or reconsolidation of memory traces. Indeed, there is initial evidence from fear conditioning research supporting this assumption^8^. However, to the best of our knowledge, this phenomenon has not been investigated so far in the context of Pavlovian reward learning, even though it may be highly relevant with respect to models of cue exposure in addiction. Taken together, we expected a smaller decrease (and potentially even an initial increase, if heightened attention may play a modulating role) in differential CRs during extinction in TC across measures. At a more exploratory level, we were also interested in the concordance of CR measures between TC and DC, as this may be informative regarding both test-retest reliability and convergent validity of conditioning tasks (which are often tacitly assumed but rarely empirically investigated^41,42^).

## Results

### Acquisition

*Self-report*. Irrespective of the *Temporal Contiguity* of US administration (*trace* vs. *delay*), participants rated the expectancy of monetary rewards after conditioning significantly higher for the CS+ (*M* = 66.9%, *SD* = 17.0) than the CS- (*M* = 17.0%, *SD* = 16.7; *t*[167] = 15.79, *p* < .0001, *d*_*z*_ = 2.02). No other effects approached significance (all *F*s < 1, *p*s > 0.37). Moreover, the CS + was perceived as significantly more pleasant (CS+: *M* = 0.613, *SD* = 0.157; CS-: *M* = 0.489, *SD* = 0.160; *t*[167] = 5.68, *p* < .0001, *d*_*z*_ = 0.75) and more arousing (CS+: *M* = 0.481, *SD* = 0.176; CS-: *M* = 0.381, *SD* = 0.178 ; *t*[155] = 4.61, *p* < .0001, *d*_*z*_ = 0.55), which did not differ between TC and DC either (all *F*s < 1, *p*s > 0.49).

**Fig. 1:**
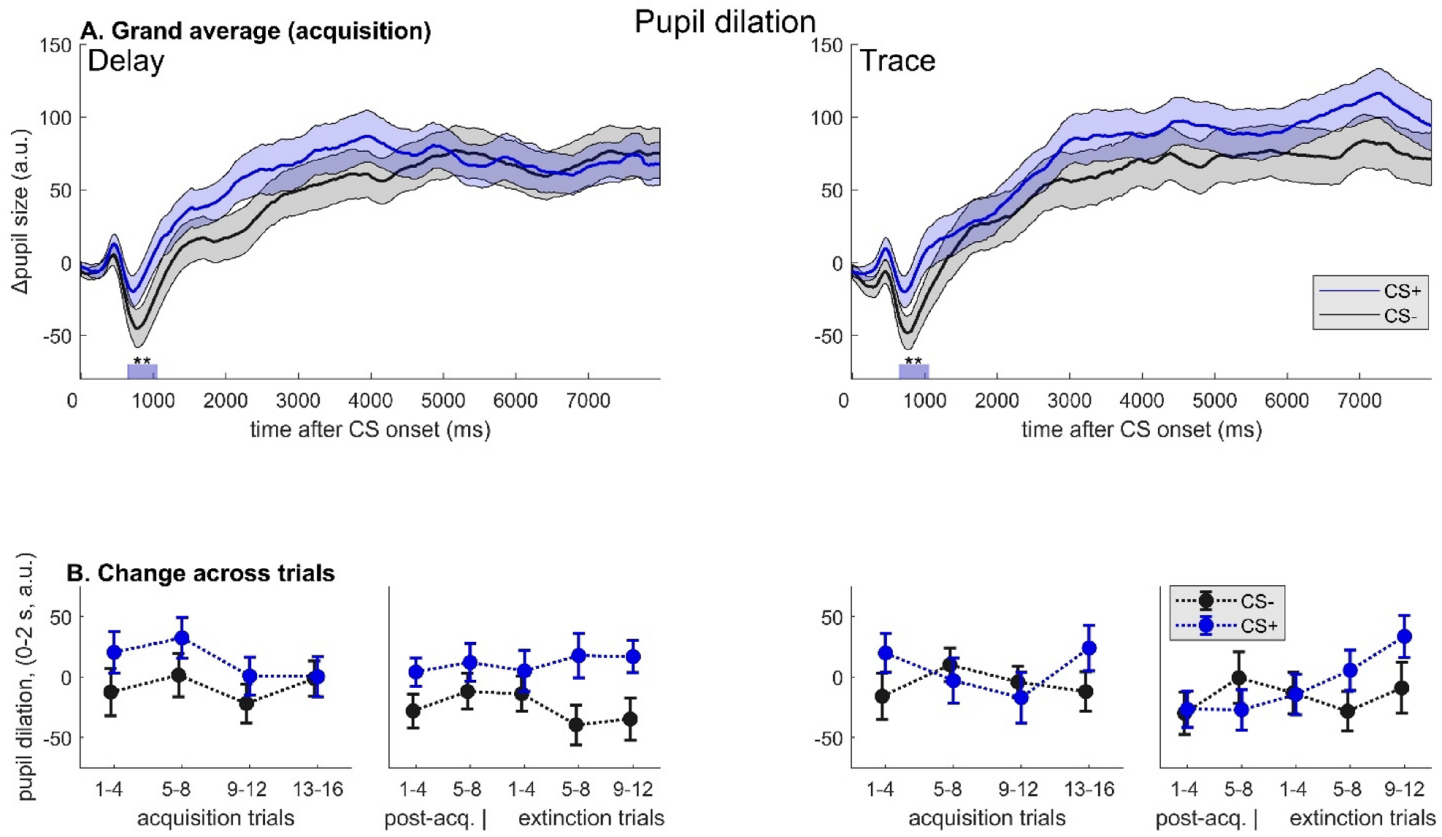
Pupil dilation responses. **A** Grand averages of changes in diameter after cue onset during acquisition phase, **B** changes in early pupil dilation (0-2000 ms after CS onset) across experimental phases/trials, grouped by timing of the unconditioned stimulus (left panel: delay, right panel: trace conditioning session). Note. Thin lines/error bars: *M*±*SEM*. CS+: conditioned stimulus; CS-: never reinforced control stimulus. Asterisks indicate significant differences between conditions (CS type) during acquisition as shown by cluster-based permutation testing (CS+ > CS-, *p* < .01). Note that, in contrast to Figure 3, all plots are based on the complete sample included in acquisition analyses.

*Physiological markers*. As shown by permutation testing across the whole CS interval, PD was significantly greater during presentation of the CS + compared to the CS- in a cluster ranging from 700 to 1040 ms after CS onset (*p* = .008, *d*_*z*_ = 0.44), irrespective of *Temporal Contiguity*, whereas other clusters did not approach significance (see Fig. 1A for illustration). Correspondingly, mixed-model analyses revealed a significant effect of *CS Type* (*F*[1,3196] = 5.96, *p* = .015) for early PD (0-2000 ms after CS onset), yet not for late PD (6000–8000 ms: *F*[1,3204] = 0.65, *p* = .422). Notably, fixed effects of both *CS Trial* and *Temporal Contiguity* (as well as the *CS Trial × CS Type* interaction and all other interaction terms) were non-significant and negligible in size (early PD: all *F*s < 1, *p*s > 0.46; late PD: main effect *Temporal Contiguity*: *F*[1,3204] = 2.97, *p* = .085; all other *F*s < 1.2, *p*s > 0.28), indicating (1) that the type of conditioning did not play a role for the acquisition of early differential pupil responses and (2) that response differentiation in this measure was relatively stable across the acquisition phase. Although the interactions were not significant, visual inspection shows that the largest differences were found during the first half of acquisition, particularly in the DC condition. See Fig. 1 (B).

**Fig. 2:**
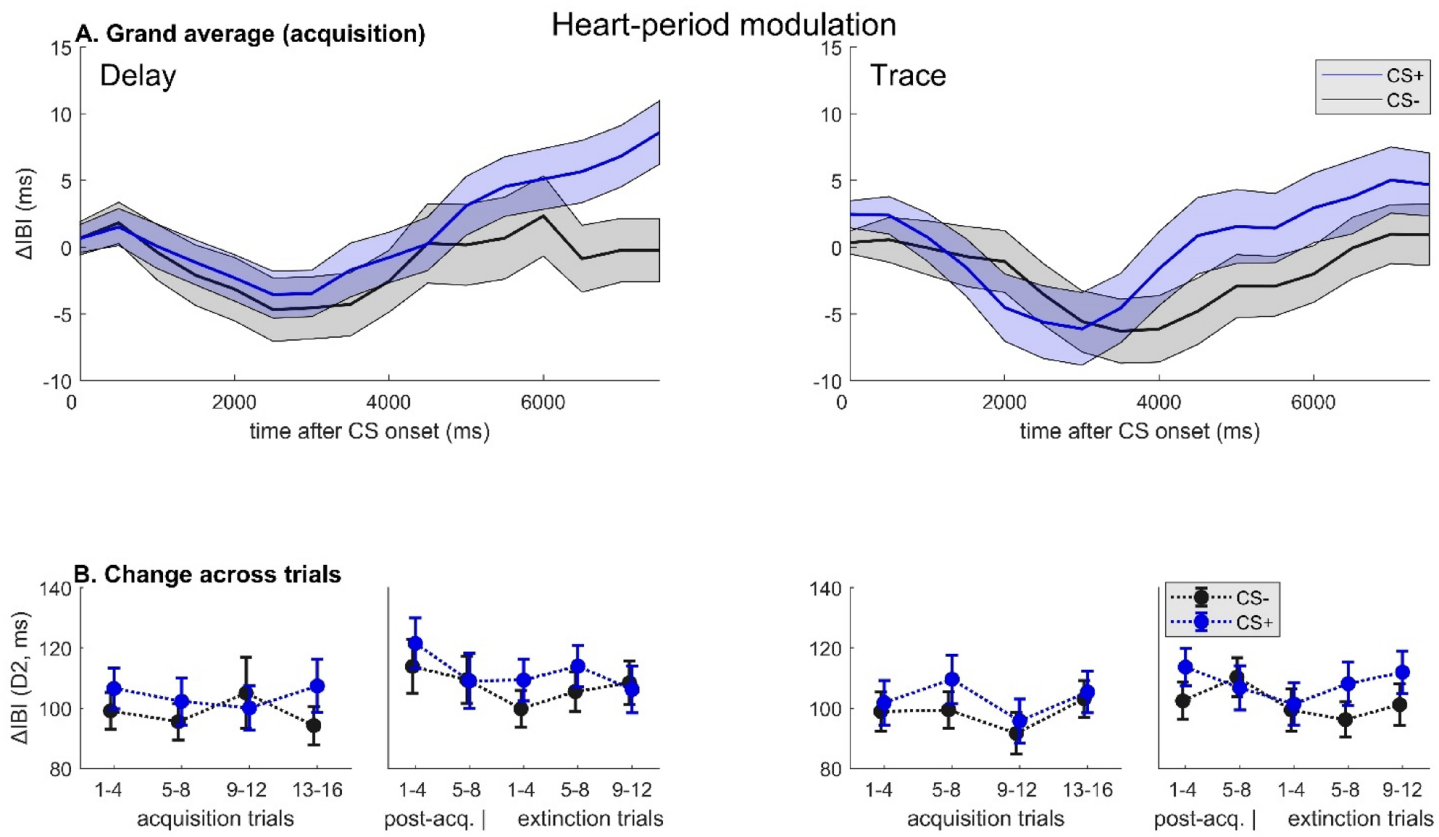
Heart-period responses. (**A**) Grand averages of changes in inter-beat interval after cue onset during acquisition phase, (**B**) changes in D2 component (second deceleration) across experimental phases/trials, grouped by timing of the unconditioned stimulus (left panel: delay, right panel: trace conditioning session). Note. Thin lines/error bars: *M*±*SEM*. CS+: conditioned stimulus; CS-: never reinforced control stimulus; IBI: inter-beat interval. Note that, in contrast to Figure 3, all plots are based on the complete sample included in acquisition analyses.

With respect to HP modulation, a triphasic response pattern emerged (consistent with previous appetitive as well as aversive conditioning studies^29^; see Fig. 2A for illustration). Maximum deceleration of inter-beat intervals (D2 component) was found to be significantly larger during presentation of the CS+ (*M* = 103 ms, *SD* = 43.9) as compared to the CS- (*M* = 97.9 ms, *SD* = 35.1; *F*[1,3473] = 4.29, *p* = .038; *d*_*z*_ = 0.24), whereas, similar to PD, no other effects approached significance (all *F*s < 1, *p*s > 0.5). See Fig. 2 (B).

Startle responses (elicited during post-acquisition) were modulated by an interplay of *CS Type* and *Temporal Contiguity* (*F*[1,1196) = 6.02, *p* = .014; interaction term: *b* = 0.288, *SE* = 0.117, *t*[1251] = 2.45, *p* = .014; main effect *CS Type: b* = -0.152, *SE* = 0.082, *t*[1251]= -1.85, *p* = .065; *Temporal Contiguity*: *b* = -0.154, *SE* = 0.084, *t*[1251]= -1.84, *p* = .066; intercept: 0.365, *SE* = 0.059). While attenuation of the ASR during presentation of the CS+, relative to the CS- (i.e., CS- > CS+), was marginally significant after DC (*p =* .065; *d*_*z*_ = 0.24), this was not the case for the difference observed after TC which showed the opposite pattern (*p* = .104, *d*_*z*_ = -0.23). Note that neither of these contrasts would survive a (Bonferroni-Holm) correction for multiple comparisons, i.e., there was no conclusive evidence for startle modulation after acquisition in either group. (See supplement for a graphical illustration of ASR results.)

Taken together, we found evidence for equivalent levels of learning regarding self-report measures as well as significant differentiation (CS + vs. CS-) of psychophysiological responses in both TC and DC. No modulatory effects of the type of conditioning (*Temporal Contiguity*) emerged, except for ASR attenuation (with only DC showing a trend in the expected direction). Interestingly, there was no indication of any substantial associations between differential CRs in DC and TC for any measure (with non-significant Spearman *ρ*s ranging between − 0.1 and 0.2; see supplement for detailed correlational results.)

### Extinction training

For those participants who showed evidence of contingency learning as indexed by self-report (*N* = 40, i.e., 71.4%, see Method), we compared the extent of (partial) extinction of differential CRs between the TC and DC sessions.

**Fig. 3:**
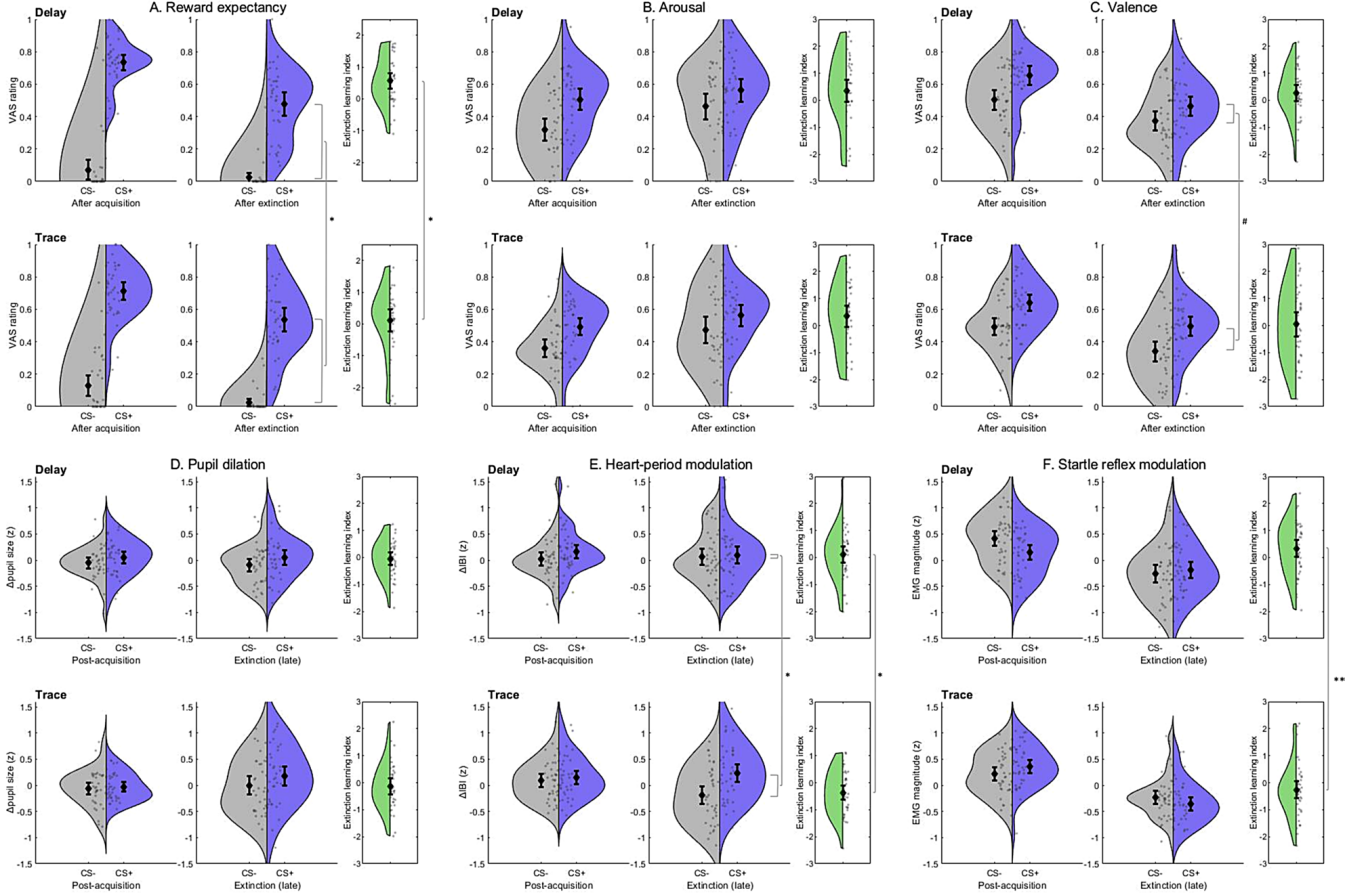
Comparison of effects of extinction training on differential conditioning indices (mean responses to the CS + vs. CS-) between delay and trace conditioning. **A**–**C**. Self-report indices assessed immediately after acquisition vs. extinction training. D.-F. Physiological markers recorded during the post-acquisition phase vs. late extinction (i.e., average responses on the last four trials per CS). Note. Error bars: 95%CI. CS+: conditioned stimulus; CS-: never reinforced control stimulus; IBI: inter-beat interval; VAS: visual analogue scale. For reasons of clarity and comparability, responses of all physiological variables were converted to z-scores (within participants/sessions), and values of the extinction learning index for startle responses have been reverse-coded to reflect startle attenuation. Levels of significance: ** *p* < .01; * *p* < .05; # *p* < .10.

*Self-report.* After extinction training, differential reward expectancy varied significantly between the *trace* and *delay* conditions (*t*[36] = 2.60, *p* = .013, *d*_*z*_ = 0.43), with larger residual differences (CS + > CS-) in expectancy following TC. Moreover, while differential valence (*t*[36] = 1.73, *p* = .092, *d*_*z*_ = 0.28) and arousal ratings (*t*[36] = 0.27, *p* = .79, *d*_*z*_ = 0.04) were not significantly different, there was an effect of *Temporal Contiguity* on double difference scores (‘extinction learning index’, ELI) of reward expectancy as well (*t*[36] = 2.40, *p* = .022, *d*_*z*_ = 0.40), indicating a more pronounced decrease in expectations during the DC session. Note that these effects remain significant after correction for multiple comparisons. See Fig. 3 (A-C).

*Physiological markers.* Similarly, end-point differences (CS + vs. CS-) in HP modulation (*t*[38] = 2.47, *p* = .018, *d*_*z*_ = 0.40) varied between TC and DC, again with larger differential responsiveness in the *trace* condition. Consistently, extinction-related reductions (ELI) in differential HP responses were greater during the DC session (*t*[﻿38] = 2.31, *p* = .026, *d*_*z*_ = 0.37). (See also Fig. 2B for changes in HP modulation across experimental phases.) An equivalent result was found for changes in ASR attenuation (ELI difference: *t*[39] = 3.30, *p* = .002, *d*_*z*_ = 0.52), even though differential responsiveness (CS + < CS-) during the last extinction trials did not significantly differ between TC and DC (*t﻿*[39] = 1.55, *p* = .128, *d*_*z*_ = 0.25). Note that effects on HP and ASR remain significant after correction for multiple comparisons. By contrast, there was no effect of *Temporal Contiguity* on differences in PD at the end of extinction training (*t*[35] = 0.20, *p* = .85, *d*_*z*_ = 0.03), and ELI scores were not significantly different for any other CR measure (all *t*s < 0.9, *p*s > 0.37). (See also Fig. 1B for changes in PD across experimental phases.) Notably, however, in the *trace* condition differential CRs after extinction training were consistently more pronounced for every measure (in numerical terms), in conjunction with lower ELI scores, suggesting a largely coherent overall pattern. See Fig. 3D-F.

## Discussion

In learning, timing matters, yet there is limited evidence on the role of temporal contiguity between CS and US for the acquisition as well as extinction of CRs in appetitive Pavlovian conditioning. Using monetary rewards as reinforcers and a wide range of measures of conditioned responding, the present study provides the first detailed within-subject comparison between appetitive TC and DC in humans, supporting reliable conditioning effects in both types of learning. During the initial learning phase, no differences between the two conditions emerged in terms of self-report as well as most psychophysiological measures (contrary to tentative hypotheses), with several measures showing substantial effects of the CS-US contingency on response differentiation (ranging from medium to large [self-report ratings] and small to medium [physiological markers], respectively). Thus, in line with classical findings from trace eyeblink conditioning research^14^, outward patterns of CR acquisition do not appear to differ between appetitive DC and TC (with short trace intervals) in aware individuals (despite presumed differences in basic learning mechanisms^4,15^). While the acquisition of conditioned PD and HP responses was not affected by the presence (or absence) of a 4-s trace interval at all, there was, however, evidence for diminished, and possibly even inverted, ASR modulation in TC (as compared to DC). In contrast, the degree of CS-US contiguity (during acquisition) appeared to have noteworthy effects on extinction learning: In TC, both differential reward expectancy and HP modulation showed evidence of a blunted decrease in CRs following extinction training, with the finding of relatively increased startle attenuation also consistent with this pattern of results (note, however, that the response difference between conditions during post-acquisition limits the comparability of ASR findings to a certain extent). Notably, the results of several other outcome variables showed similar numerical trends (yet without statistical significance). To our knowledge, this is the first human study providing evidence for reduced extinction following appetitive TC, which has previously only been documented with respect to fear conditioning^8^.

In line with previous reports, we observed increased PD^24,25,43^ as well as HP deceleration^24,29^ during presentation of reward-predicting CS in the acquisition phase, indicating that both measures are sensitive physiological markers in both DC and TC. Although there was no statistical evidence of a change in response differentiation across acquisition trials, one way of interpreting the current data would be that learning was rapid (i.e., occurred within the first few trials) and that discrimination then slowly declined. With respect to conditioned PD (early response phase), a particularly high level of stability of differential CRs after extinction training, irrespective of CS-US contiguity, was evident (late extinction: *d*_*z*_ = 0.22), suggesting a lower susceptibility to extinction effects. This is in line with previous studies that showed little or no habituation of conditioned PD^26,44^ as well as persistent differentiation at delayed recall^44,45^, pointing to early-phase PD as a potentially long-lasting index of contingency learning. However, it must also be noted that, in contrast to previous findings from fear conditioning research (e.g^46^). , as well appetitive conditioning using primary reinforcers^24^, no effect was observed in the late response window (preceding US onset in DC) – although this is in fact consistent with earlier work from our own group using monetary rewards^25^. Overall, PD results fit with the notion that PD may capture specific aspects of the learning process which are not reflected in other measures, such as the engagement of attentional resources due to uncertainty and prediction-error processing^47^. In contrast to associative strength (i.e., expected value), learning parameters thought to reflect uncertainty about outcomes may increase transiently during initial extinction training, providing a potential, tentative explanation for the persistent pupillary effects. This interpretation (in terms of attentional effects) would also be consistent with the observed numerical pattern of PD changes across trials, which suggests that both conditions showed evidence of rapid learning within the first few acquisition trials, followed by a plateauing or even slightly decreasing trend. In DC, ASR results during post-acquisition were largely in line with expectations, showing a valence-dependent modulation at trend level^31^. However, this was not the case for TC, pointing to either blunted processing of CS valence in TC (which would be at odds with the results of valence ratings) or other alterations in affective learning processes, compared to DC. In our view, the most likely explanation for the observed dissociation is that startle attenuation by concurrent appetitive cues may be contingent on the expectation of imminent US delivery, rather than on the affective value of the CS per se. In fact, previous appetitive^31,32^ as well as aversive conditioning studies (e.g^48,49^). that assessed ASR modulation elicited startle responses during a time window shortly before (expected) US onset (including trace fear conditioning studies^6^), which is consistent with this notion. By contrast, cues linked to delayed US expectation may be processed in terms of their predictive (rather than affective) value, and potential startle facilitation in TC may be explained by increased engagement of attentional processes, known to increase startle responsiveness at long lead intervals^50–52^ (even with affectively positive content^53^). While we cannot address this tentative hypothesis on the basis of the present data alone, it certainly merits further attention in future research. Notably, we did not observe any effects of appetitive conditioning on SCRs (see Methods), which have previously been found to be very sensitive to subtle differences in contextual and task-related features^54^ and often appear to yield null findings in the context of appetitive (as opposed to aversive) conditioning^25,29,55^. At an anonymous reviewer’s request, we performed an additional analysis to check for effects of sequence (TC on the first vs. last session) as well as position (session number) on all outcome measures, revealing that on the second session participants’ post-acquisition ratings discriminated more strongly between CS + and CS-, in terms of both US expectancy (*p* < .001) and valence (*p* = .042), than after the first visit to the lab, presumably reflecting a ‘practice’ or nonspecific carryover effect. Crucially, there was no effect of sequence and no further interactions (*p*s > 0.37), and differential conditioning of physiological measures was not modulated by position or sequence at all (all *p*s > 0.13). Therefore, this effect is unlikely to substantially alter the interpretation of the results.

There are several potential explanations for the differential extinction effects observed in our study: (1) While neither cognitive contingency learning nor the acquisition of differential CRs at the physiological level seemed to be more difficult overall in TC, it is conceivable that the reduced temporal contiguity nevertheless induced heightened perceptions of unpredictability, similar to effects of partial reinforcement seen in both Pavlovian and operant conditioning^36,39^. Diminished extinction of reward expectancy in TC would thus be in line with the established view that unpredicted rewards engage the dopaminergic system more strongly^56^, which may contribute to slower learning of changed reward contingencies (as seen in addiction). (2) Relatively stronger engagement of brain systems linked to the cognitive processing and prediction of stimulus-outcome contingencies, such as the prefrontal and anterior cingulate cortex, along with structures involved in contextual memory encoding, such as the hippocampus^4^, may facilitate an overall deeper encoding of memory traces during acquisition which are then more difficult to override by extinction learning. (3) Another related possibility is that the memory systems underlying DC are more susceptible to suppression by novel, inhibitory memory traces formed during extinction learning^57^. While it is important to note that these tentative interpretations are not mutually exclusive, nor do the present data allow to draw any firm conclusions that would definitively rule out any of these alternatives, these considerations do point to potential, plausible pathways that could explain our results. Nevertheless, further research is needed to clarify the exact mechanisms underlying our findings and to disentangle the contribution of the various pathways outlined above. When interpreting the present results, it is also important to note that there is no consensus in the literature as to whether differences in CR acquisition and extinction observed between TC and DC rely on qualitatively different neural mechanisms or reflect quantitative differences in learning difficulty instead (e.g^11^). Also, many human neuroimaging studies have revealed a considerable overlap in the neural substrates of learning during TC and DC (e.g^7,12^). , .

At an exploratory level, we were also interested in the degree of concordance between the two types of learning. Strikingly, however, no intraindividual associations between TC and DC sessions were found whatsoever (with respect to all the indices of differential conditioning assessed in our study; see supplement for details). This total lack of concordance (despite about 64% power to detect Spearman correlations ρ ≥ 0.3 and 98% power for correlations ρ ≥ 0.5) suggests at least three different (again not mutually exclusive) interpretations: (1) a greater role of state, as opposed to trait, factors in differential conditioning variability, (2) systematic differences in learning processes between appetitive TC and DC^4^, and/or (3) a particularly low level of test-retest reliability of the general conditioning paradigm used in our study. Given the substantial covariation in overall CS responsiveness found for several measures, as well as satisfactory levels of reliability in fear conditioning studies based on similar designs^41,42^, the latter explanation (in terms of high unsystematic measurement error) does not seem very likely to us. Rather, we assume a combination of state factors and basic (qualitative or quantitative) differences in learning processes as the most probable account of this finding. This would be in line with a large body of research in fear as well as trace eyeblink conditioning that has documented several disanalogies between TC and DC^7,8,10^. Moreover, lesion research in rodents has challenged the assumption that appetitive TC relies on similar, hippocampus-based learning mechanisms as aversive TC^19,20^, pointing to specific, valence-dependent variation in learning processes as well.

Of course, our study has some limitations. First, in the present design CS duration was kept equal across both learning paradigms, resulting in a longer CS-US onset delay in TC than DC (which has to be bridged by memory in addition to the trace interval per se). Therefore, it cannot be ruled out that a different pattern of results may have emerged if the trace interval would instead have been formed by shortening CS presentation. Also, it is not clear whether and how the extinction-related differences in psychophysiological learning indices as well as self-report ratings might translate to actual human behavior. Moreover, while aimed at enhancing the sensitivity to variation in partial extinction of CRs, the study design did not allow to infer whether DC and TC differ in the speed and trajectory of acquisition as well as extinction learning. For instance, it remains unclear whether any differences observed between the two types of learning would still exist after a prolonged (full) extinction phase. We also did not examine the role of contingency awareness in TC, but rather controlled for this potentially confounding factor in the analyses of extinction learning by excluding the (rather small) fraction of participants who had not acquired explicit knowledge of contingencies. In addition, future research should address the relevance of US type, for example, by comparing effects of primary vs. secondary reinforcers, as well as potential dose-response characteristics. This is particularly relevant as the amount of money administered as US in the present study was rather small (at least at the level of single trials), raising the question whether stronger conditioning effects or even a somewhat different pattern of results would have emerged with a more potent (e.g. primary) reinforcer. Another aspect that may have influenced the present results, including the outcome of the DC-TC comparison, is the partial contingency instruction used in our study. Although this is an established approach in human conditioning research^23^, in order to facilitate learning while reducing inter-individual variability in contingency awareness, it is possible that TC may have benefitted more than DC from the prompt to look for a relationship between stimuli, thereby disproportionately increasing the number of participants who became aware of contingencies in TC. This, in turn, may constrain the generalizability of our findings to some extent.

In conclusion, our study extends existing evidence for differences between TC and DC regarding underlying mechanisms to the context of reward learning. Despite similar overall patterns of CR acquisition, our findings suggest that TC might be linked to reduced appetitive extinction across several subjective as well as physiological markers of differential Pavlovian conditioning. As temporal gaps between cues and rewarding outcomes regularly occur in natural settings, these results – pending replication – might have important implications not just for theoretical models of associative learning per se, but also for understanding the pathogenesis and neurocognitive underpinnings of addiction and related maladaptive behaviors linked to appetitive conditioning^18,58^. Moreover, future research may also help to explain altered learning mechanisms that emerge in disorders such as psychosis which has been suggested to be specifically associated with changes in appetitive TC^59^.

## Methods

### Participants

In terms of assessing differential CRs (CS + vs. CS-), the present study was designed for detecting effects of at least *d*_*z*_ = 0.4 with 90% probability (corresponding to average effect sizes in appetitive conditioning studies using PD^26^). The software G*Power (Version 3.1.9.4; 60) was used for a-priori power analysis, yielding a required sample size of *N* = 55 participants for a directional hypothesis (CS + > CS-). To account for attrition, especially drop-out after the first session, we oversampled slightly, i.e., a total of 62 participants (students at the University of Siegen) were initially recruited. Sixty participants finished both sessions of the experiment, providing data for all or most outcome measures (final sample: *N* = 60, 50 women, mean age [years] = 22.0, *SD* = 3.21). After the exclusion of individual datasets (participants/sessions) containing high amounts of invalid responses (e.g., physiological artifacts; see below), *n* = 58 (48 women) full datasets of both sessions remained for analyses of PD during the acquisition phase (HP: *n* = 60 [50 women], ASR: *n* = 57 [48 women]). Because of technical problems (computer failure), self-report data were completely lost for 4 participants (one session), as were self-report and pupillary data recorded during extinction training for another 4 participants (at least one session) who could thus not be included in the analysis of extinction learning. Moreover, to rule out that the comparison between TC and DC was biased by prior differences in acquisition, only participants showing clear evidence of discriminatory learning regarding the CS + in both sessions (as indexed by self-report; see below) were included in analyses targeting differential extinction effects (*N* = 40 [33 women], mean age [years] = 21.55, *SD* = 2.52). Sensitivity analyses showed that sample sizes for assessment of CRs during acquisition (*n* = 57 to 60) allowed to detect small to medium-sized effects (interactions with *Temporal Contiguity*) around *f* = 0.17 to 0.18 (with 90% probability). In the context of extinction, our study was sensitive to medium-sized effects around *f* = 0.21.

A-priori criteria for eligibility were no intake of psychotropic medication and no mental health problems during the last two years, no tinnitus or hyperacusis and no evidence of acute or severe, chronic somatic disorders. Written informed consent was obtained from all participants before the start of the study. Participants were recruited via mailing lists and flyers at the university and contacted beforehand via phone in order to ascertain criteria for eligibility and schedule an on-site appointment. After study completion, participants received the full amount of money they had won during the conditioning task (18 €) as well as course credit as compensation. The study protocol was approved by the institutional ethics committee (‘Rat für Ethik in der Forschung der Universität Siegen’; LS_ER_29_2023) and adhered to the guidelines of the Declaration of Helsinki.

### Design and procedure

#### General procedure

Participants visited the laboratory on two separate days, with the interval between visits ranging from 2 to 7 days (*M* = 5.4, *SD* = 2.1). Upon arrival at the laboratory on their first visit, participants were first informed about the general methods and procedures employed in the study. After signing the informed consent form, they filled in a short questionnaire about demographic and health-related information. Then, they were seated at a desk in the testing chamber (in a different room). After the experimenter had attached electrodes for psychophysiological measurements, the eye-tracker was calibrated. Then, participants performed the first conditioning session which lasted approximately 40 min (including break and average time taken for ratings). The second session was identically structured (apart from initial instructions, signing of informed consent and questionnaire). Participants underwent either the DC session first and then the TC session or vice versa (counterbalanced assignment). Immediately before each conditioning phase, participants received written instructions on the screen. Participants were told that they would be presented with a series of visual stimuli which were either followed or not followed by a display of an amount of money they had won, and that they would have to figure out the relationship themselves (i.e., partial contingency instruction). They were assured that they would be able to keep all the money they won which would be paid out at the end of the session. After each conditioning session (i.e., after extinction training), participants received 9 € in cash. At the end of the second session, they were thanked and fully debriefed.

**Fig. 4:**
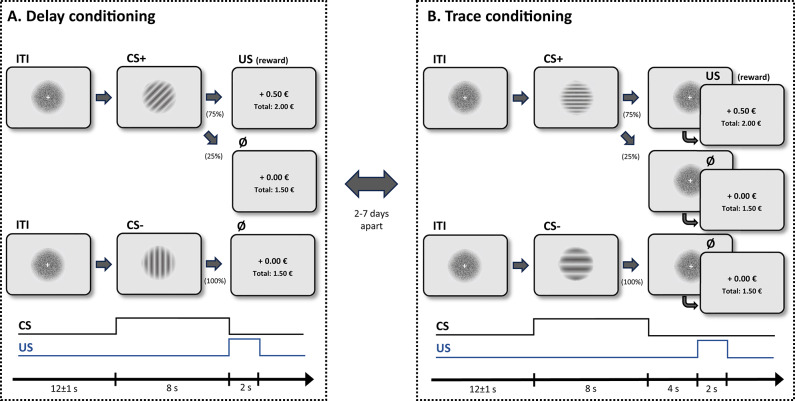
Design and trial structure (acquisition phase). *Note* CS+, conditioned stimulus (followed by an unconditioned stimulus in 12 of 16 trials during acquisition); CS-: never reinforced control stimulus; US: unconditioned stimulus (monetary reward); ITI: inter-trial interval.

#### Acquisition

 The experiment started with a brief habituation phase, containing 2 CS + and 2 CS- trials. Habituation trials had the same structure as acquisition trials, but were not reinforced at all. During the subsequent acquisition phase, in both conditioning sessions, each CS (i.e., CS + and CS-) was shown 16 times for a duration of 8 s in the middle of the screen (see Fig. 4 for illustration). Different sets of stimuli were used for the two sessions (counterbalanced assignment). The order of trials was fully randomized, apart from the following constraints: (a) The first two trials contained stimuli from both CS conditions; (b) the first CS + trial was always reinforced; (c) a particular CS did not appear on more than two successive trials. In the DC session, a slide with the text “+ 0.50 €” (US) as well as the updated “total” account value (see Fig. 4) was shown immediately after offset of the CS + for 2 s in 75% (i.e., 12/16) of trials (before the start of the inter-trial interval, ITI). In unreinforced trials (including all CS- trials) “+ 0.00 €” (non-US) was shown instead. The procedure in the TC condition was identical, apart from the fact that the US/non-US was presented after a delay of 4 s, during which the ITI slide was shown. The ITI (after US/non-US offset) lasted for 12 ± 1 s (random jitter) and consisted of the presentation of a perceptually matched control slide (see Stimuli and apparatus), in order to control for physical influences on pupil size and prevent activation of the light reflex of the pupil during CS onset. See Fig. 4 for an overview of the design of trials in each condition.

After acquisition, participants were prompted to provide ratings of subjective arousal, valence and reward expectancy for the CS + and the CS- (see below).

#### Extinction training

 After a break of 3 min, the experimenter entered the room and attached EMG electrodes at the right m. orbicularis oculi as well as headphones for measuring/eliciting startle eyeblink responses. Then, the eye-tracker was re-calibrated, and participants were told (via written instructions on the screen) that they would again be presented with a series of visual stimuli and, this time, with occasional bursts of loud noise as well that could be ignored. After the presentation of a sequence of six such startle probes with a latency of 6–10 s (aimed at startle habituation), the second part of the conditioning session started with a block of partially reinforced trials (‘post-acquisition phase’): During this phase, the two CSs were again shown 8 times each with the same duration, trial structure and reinforcement rate as before (i.e., CS + presentation was followed by a monetary reward of 0.50 € in 6 cases). Then, without noticeable change, 12 completely unreinforced trials (per CS) followed (‘extinction phase’). Again, the order of trials was fully randomized, apart from the constraints as already mentioned above. Acoustic startle probes (white noise) were always presented on the first trial (per CS) and in 75% of trials overall during both post-acquisition and extinction (in randomized order, counterbalanced across stimuli and conditions, at a delay of 5–6 s relative to CS onset).

After extinction training, participants were again prompted to provide ratings of subjective arousal, valence and reward expectancy.

### Stimuli and apparatus

#### Conditioned stimuli

 Greyscale, circular grating stimuli (see Fig. 4) varying either in orientation (rotation angle; set 1) or in grid width (spatial frequency; set 2) were used as CSs. For each participant the two different stimulus sets were assigned to the DC and TC sessions, respectively, in a pseudorandomized, counterbalanced way. Also, the assignment of individual stimuli (within each set) to the type of CS (CS+, CS-) was randomly varied between participants. All stimuli were shown in the center of the screen in front of a light-grey background, subtending a visual angle of approximately 5° × 5°. A control slide matched to CSs in terms of brightness, contrast and dispersion was presented during the ITI, consisting of a circle filled with randomly scattered pixels as well as a small white fixation cross.

#### Apparatus

 Participants were seated in front of a 27-inch TFT screen (with a resolution of 1920 × 1080 pixels and a refresh rate of 60 Hz) at a viewing distance of 70 cm, with their forehead and chin resting on a head rest mounted on a height-adjustable desk. The experiment was programmed with the Psychtoolbox (Version 3.0.10;^61^) implemented in MATLAB (MathWorks, Inc., Natick, MA, USA) and ran on a standard Windows PC.

#### Acquisition of physiological data

 Pupil diameter was recorded from the right eye at a sampling frequency of 1000 Hz using a video-based infrared eye-tracking device (EyeLink 1000 Plus, SR Research Ltd., Ottawa, Canada). Calibration of the eye-tracker was performed by means of an in-built 9-point calibration routine and repeated until an accuracy of < 1.0° was achieved.

Electrocardiographic (ECG), electromyographic (EMG) and electro-dermal activity (EDA) was continuously recorded by means of a Biopac MP160 recording system (Biopac Systems, Inc.) with 16-bit resolution and 2000-Hz sampling rate. Electrodes for ECG measurement (Biopac EL-503 were attached according to a standard lead II configuration (proximal electrode placement on the chest). The raw ECG signal was high-pass filtered (0.5 Hz, Biopac ECG100). Cup Ag/AgCl electrodes for EDA measurement filled with isotonic NaCl gel (with a conductive area of 8 mm diameter) were placed at the non-dominant hand (thenar/hypothenar). EMG activity of the right orbicularis oculi muscle was recorded unilaterally via Kendall Healthcare H124SG Ag/AgCl electrodes (conductive area: 8 × 8 mm; electrode distance: about 1.5 cm) with bandpass filtering (28–500 Hz), rectification, and integration (time constant: 10 ms). Startle eyeblink responses were elicited by short binaural bursts (50 ms) of white noise at 103 dB(A) SPL.

### Data reduction and analysis

#### Self-report ratings

 Immediately after the acquisition phase as well as after extinction training, participants were prompted to indicate the expectancy of a monetary reward for each CS using a visual analogue scale (VAS) ranging from 0 to 100% (probability of occurrence). Subjective levels of arousal and valence attributed to each CS were assessed via VAS as well, with the anchors ‘calm and relaxed’ to ‘very arousing/tense’ and ‘very unpleasant’ to ‘very pleasant’, respectively. Distances in pixels were converted to arbitrary units (ranging from 0 to 1).

#### Pupil dilation (PD)

 Preprocessing of pupillometric data was performed in MATLAB (MathWorks, Inc.), in accordance with recently published recommendations^62^ as well as procedures established by prior conditioning studies^25,44,63^. After segmentation into trial epochs and down-sampling to 100 Hz (by means of a moving window filter selecting the median value across 10-ms epochs), missing data points (resulting from blink artifacts and small head movements) were replaced by linear interpolation (6.6% of total data recorded during acquisition in the final sample). On average, 1.7 trials per participant and session were rejected (i.e., 5.2%, *SD* = 8.1). Individual datasets (sessions) with less than 8 valid trials (i.e. 50%) per CS were not included in further analyses (see Statistical analyses, below). Specifically, invalid trials were defined as (i) trials during which eye gaze was not directed at the center of the screen (e.g., due to prolonged blinking, distraction etc.) for at least 50% of CS duration, based on a cut-off window around the participant’s median gaze position (across all trials) covering approximately ± 5° visual angle (0.75% on average in the final sample, *SD* = 2.2), (ii) trials with high amounts (> 50%) of interpolated data (2.8% on average, *SD* = 5.1) as well as (iii) trials containing values exceeding a threshold of 3 SDs above or below the participant’s mean pupil size (1.0%). After data cleaning, pupil traces of each trial were baseline-corrected by subtracting the mean pre-stimulus value (averaged over a period of 500 ms immediately before CS onset) from each subsequent data point.

In accordance with previous research^25,26^, we extracted mean change scores (per trial) for an early (0-2000 after CS onset) and a late (0-2000 before US onset, i.e., 6000–8000 after CS onset) response interval for statistical analyses. To further validate this analytical strategy by means of a more data-driven, yet at the same time conservative approach, stimulus-locked time series of pupillary waveforms across the whole time window of CS presentation (downsampled to 100 Hz and averaged by participants and experimental conditions) were additionally analyzed by means of a cluster-based permutation ANOVA (using the Matlab-based function *fctSnPMS.m*;^64^). By summing up clusters of adjacent values above a certain threshold of significance (*F* values with *p* =. 05 in this case) and testing this index against a distribution of randomly permutated data, this non-parametric approach allows for preserving adequate statistical power while effectively controlling for alpha error inflation in the analysis of complex data with multiple sample points. 1000 permutations were generated for assessing main effects and interactions (of *CS Type* and *Temporal Contiguity*).

#### Heart-period (HP) response

 QRS complexes were identified offline in the ECG signal using the Pan-Tomkins algorithm. The signal was carefully inspected for epochs contaminated with technical or physiological artifacts (including premature heart beats) and manually corrected, if possible. In total, 2.3% of trials were discarded due to high amounts of artifacts or missing data (*SD* = 5.3%). After down-sampling (100 Hz), inter-beat intervals (IBI) were baseline-corrected for each trial (by subtracting the average value across 1000 ms before CS onset) and averaged in 500-ms segments across CS duration, weighted by fractions of real time^65^. As previous conditioning studies have indicated a triphasic response pattern (consistent with grand-averages of waveforms in our data), characterized by an initial deceleratory component (D1), followed by a subsequent acceleratory component (A) and another deceleration phase (D2), these response phases were dissociated and separately extracted from the waveform of each trial. Specifically, in accordance with previous research^29^, our analysis focused on the D2 component, which was defined as the maximum HP change (in ms) before US onset, relative and subsequent to A (with A calculated as the fastest 500-ms time bin, i.e., minimum HP value within the first 5 s after CS onset and D1 as the slowest time bin during the first 2 s after CS onset^29^).

#### Acoustic startle eyeblink reflex (ASR)

 The filtered, rectified and integrated EMG signal was further processed by means of a customized C + + based program. For each trial, peak EMG activation during a time interval of 20 to 150 ms after startle probe onset was determined. Startle magnitude was defined as the difference between EMG peak and baseline amplitude (i.e., the average value across an artifact-free interval about 50 ms prior to startle probe onset). All responses were inspected manually to check for electrical as well as physiological artifacts. Trials contaminated with artifacts (e.g., immediately preceded by spontaneous blinks) were rejected from analysis and defined as missing (9.7% in the final sample; *SD* = 10.7). If there was no visible response at the typical response latency of a particular participant, response amplitude was set to zero. Zero responses (0.86% on average; *SD* = 4.2) were included in further analyses, and all response values were converted to intraindividual z-scores prior to statistical analyses (which is a best-practice approach for the analysis of startle response data due to their high levels of interindividual variability^66^).

#### Skin conductance response (SCR)

 After down-sampling to 100 Hz, low-pass filtering (5 Hz), and smoothing with a Gaussian window, raw EDA data were analyzed by means of the MATLAB-based Ledalab toolbox (Ledalab 3.4.9;^67^). After extraction of tonic and phasic components by means of standard deconvolution, average phasic driver activity during CS presentation (1000–8000 ms after onset) was used for scoring conditioned SCRs. Each individual dataset was visually inspected for artifacts. However, we refrained from subjecting SCRs to detailed statistical analyses due to a large number of individual datasets with very small proportions of valid SCRs (i.e., responses above a threshold of 0.01 µSiemens). In total, 53 participants had at least one session with more than 25% zero responses in the acquisition phase, with 40 participants being above 50% (*M* = 55.5%, *SD* = 30.6). Nevertheless, to rule out systematic differences in the distribution of valid responses between experimental conditions, we computed a generalized linear mixed-effect model with dichotomous responses at the trial level (SCR: 0 vs. 1) as dependent variable and *CS Type* (CS + vs. CS-) as well as *Temporal Contiguity* (trace vs. delay) and their interaction as fixed effects. This exploratory analysis revealed no significant effects involving *CS Type* (all *p*s > 0.3) suggesting that the conditioning task used in our study did not induce robust differentiation of SCRs in our sample. Therefore, we do not report detailed SCR results.

### Statistical analyses

#### Acquisition

 To assess successful differential conditioning as well as potential differences as a function of *Temporal Contiguity* between CS and US (trace vs. delay), CRs of all psychophysiological measures (PD, HP and ASR) were extracted at the trial level and analyzed by fitting separate linear mixed-effects models to the data. Self-report measures (post-acquisition ratings of reward expectancy valence and arousal) were analyzed in an analogous manner. Moreover, to provide further evidence regarding the temporal dynamics of conditioned PD, time series data of average pupillary traces were additionally analyzed using cluster-based permutation tests (as explained above). Linear fixed-effects models were computed using the *lme4* package available in *R*;^68^). This approach controls for clustering of related data by means of random effects (estimated variance components) in addition to fixed effects of experimental conditions^69^. For PD and HP, fixed effects of *CS type* (CS + vs. CS-), *Temporal Contiguity* (trace vs. delay) and *CS Trial* (2–16), and all interactions were evaluated, in addition to random intercepts for participants. Responses to the CS during the first trial of the acquisition phase (per CS) were excluded from statistical analyses as no associative learning (with respect to the CS-US contingency) could have occurred at that point. Conditioning effects on ASR during the post-acquisition phase (6 startle trials per CS/session) were assessed by including *CS type* and *Temporal Contiguity* as fixed effects. For all dependent variables, the initial, full model (including all fixed effects and interactions as predictors) was reduced in a stepwise, backward fashion by comparing goodness of fit of the respective full model against an incrementally reduced model (comparisons based on maximum likelihood estimation). Initially, to control for potential sequence effects, models with random slopes for number of *Session* were also tested, but since this did not lead to significantly improved model fit, yet worse fit in some instances, *Session* was not included in any final model. Significance of fixed effects was assessed by t-tests with Kenward-Rogers approximation of degrees of freedom. For all analyses, the threshold of significance was set to α = 0.05. Furthermore, statistical differences at *p* < .10 are discussed as trends. While unadjusted p-values are reported for ease of interpretation, all follow-up tests (performed in case of significant interactions) were additionally checked for multiple comparisons using the Bonferroni-Holm procedure, applied separately for data from each outcome measure. For each set of tests, we indicate in the text whether the results would survive correction.

For descriptive and exploratory reasons, we also report Spearman rank correlations between mean CR magnitudes of both sessions (testing for monotonic associations without the assumption of linearity), in order to characterize the relationship between TC and DC in a comprehensive manner and to explore the extent of concordance, i.e., potential associations vs. dissociations between the two types of conditioning. These additional analyses may be of interest in terms of issues of reliability and validity and may thus inform future research and theorizing. Participants with more than 50% missing CS + or CS- trials during the acquisition phase of any session were excluded from all analyses of the respective dependent variable (e.g., PD). As mentioned above, this led to discarding of pupillary data from two participants and startle data from three participants.

#### Extinction learning

 To quantify the extent of extinction learning, remaining differential responses (CS + vs. CS-) after extinction training were assessed and compared to pre-extinction levels of responding. For self-report measures, difference scores (CS + vs. CS-) between ratings (reward expectancy, valence, arousal) given immediately after the acquisition phase and ratings given immediately after the extinction phase were calculated. For physiological CR markers, we adopted an approach well-established in previous work on fear extinction learning (e.g^70,71^), by calculating average responses during the last four (valid) trials (per CS) of the extinction phase which were then compared with average responses during the preceding post-acquisition phase. Based on these measures, we first compared mean differences in responding to the CS + vs. the CS- after (ratings) or at the end of (physiological markers) extinction training between both types of learning (using two-sided t-tests). Second, to capture temporal changes in response differentiation as well as enhance comparability across various CR measures, a scale-free extinction learning index (ELI) was derived for each dependent variable, by first standardizing response values of each measure (within participants and sessions) and then calculating double differences in differential responding (CS + vs. CS-) between pre- and post-extinction periods^70^. As discussed in detail in the context of the diversity of potential ‘extinction retention indices’ (ETIs), this operationalization, relying on differential responses (i.e., responses expressed in relation to the CS-) as well as intra-individual scaling of responses, has the crucial advantage of controlling for overall levels of response habituation and other non-specific, potentially confounding effects^72^. ELIs of each CR measure were as well compared between the two *Temporal Contiguity* conditions by means of separate, two-sided t-tests. Again, for each outcome measure, we checked whether the significance of these comparisons would survive Type I error correction using the Bonferroni-Holm method.

To rule out that the comparison between TC and DC regarding potential differences in extinction learning was biased by any major differences in prior learning success, we ascertained that all participants included in these analyses had acquired knowledge of the correct CS-US contingency. Therefore, we excluded participants who chose the incorrect response to the forced-choice US expectancy rating of the CS+ (after acquisition), stating that the CS + was ‘never’ followed by a monetary reward (*k* = 4). In case participants answered with ‘don’t know’ instead (*k* = 17), they were included on condition that they attributed reliably higher levels of reward expectancy to the CS + than to the CS- (in dimensional expectancy ratings). This threshold was determined by computing a Bayesian credible (high density) interval for the difference CS + vs. CS- based on data of participants with correct forced-choice responses, which reflects the probability distribution of differential expectancy scores given overall correctly learned contingency expectations. As this analysis indicated a lower 95% bound of 0.237 (or 23.7%), participants who had indicated subjective uncertainty about contingencies but had differential expectancy scores above this value, i.e., above chance level, were nevertheless included in extinction learning analyses (*k* = 4). In total, data from 17 participants were excluded, with most showing an irregular learning performance in both conditions (only delay: *k* = 2; only trace: *k* = 0; both: *k* = 15). In addition, the same criterion for exclusion based on data validity was applied as for the acquisition phase (i.e., ≤ 50% missing trials per CS throughout the post-acquisition/extinction phase) to ensure a sufficiently high signal-to-noise ratio (leaving *n* = 36 participants for PD during extinction training, *n* = 39 for HP and *n* = 40 for ASR).

## Electronic supplementary material

Below is the link to the electronic supplementary material.

Supplementary Material 1

## Data Availability

All data are accessible at a public repository of the Open Science Framework (OSF), via the following link: https://osf.io/agjn7.
