# Supplementary material for: Intra-individual comparison of appetitive trace and delay conditioning in humans across acquisition and extinction
Source: Sci Rep. 2025 Jun 20;15:20156. doi: 10.1038/s41598-025-05350-0 (PMC12181268; doi:10.1038/s41598-025-05350-0)
Supplement: Supplementary file 1 — Supplementary Material 1 [file 41598_2025_5350_MOESM1_ESM.pdf]

Supplementary Material

Intra-individual comparison of appetitive trace and delay conditioning in humans across acquisition and extinction

Finke, Schippers & Klucken, 2025, *Scientific Reports*

Concordance between trace and delay conditioning sessions

As illustrated in Figure S1 (A-F), there was no evidence that differential conditioned responses (CRs) in delay conditioning (DC) were substantially associated with trace conditioning (TC) for any measure assessed in our study (with non-significant Spearman ρs ranging between -.1 and .2). However, in some instances (e.g., arousal ratings, heart-period response) substantial inter-session correlations between mean response magnitudes to the CS+ and CS- emerged. Note that mean magnitude of raw startle responses was in fact also strongly correlated within stimulus categories (CS+: $\rho = .70$, $p < .0001$; CS-: $\rho = .58$, $p < .0001$), with the lack of association of mean z-scored startle responses, as shown in Figure S1, resulting from intraindividual z-standardization (Blumenthal et al., 2005).

Taken together, this pattern of results suggests that, despite the lack of convergence in learning outcomes, much of the overall variability in CR measures can be explained by individual, possibly dispositional, differences in general cue responsiveness.

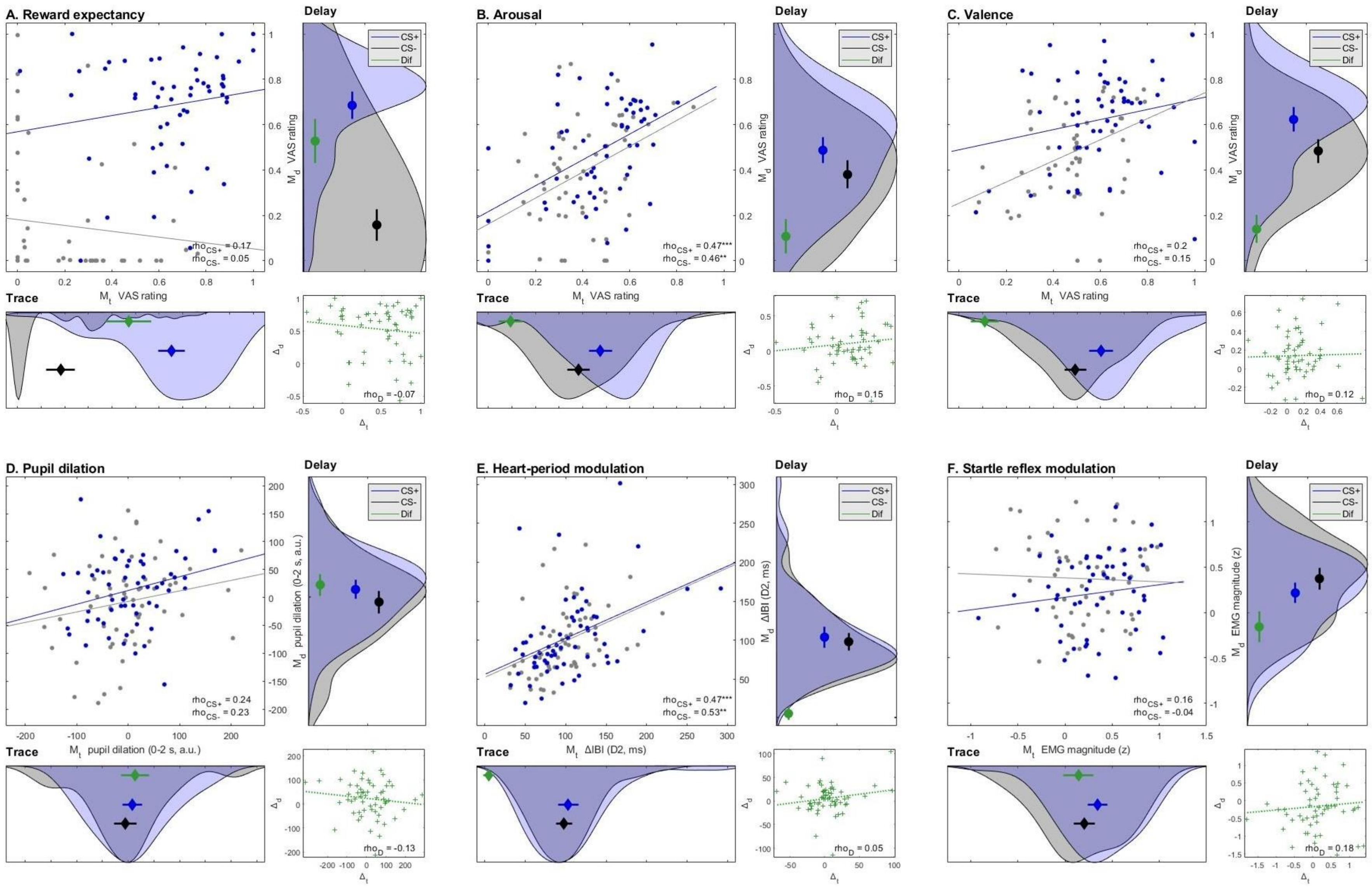

Figure S1. Distribution and correlational patterns of differential conditioning indices (mean responses to the CS+ vs. CS-, mean differential responses) in delay and trace conditioning sessions. A.-C. Self-report indices assessed immediately after the acquisition phase. D.-F. Physiological markers recorded during acquisition (D, E) or post-acquisition (E).

Note. Error bars: 95% *CI*. CS+: conditioned stimulus; CS-: never reinforced control stimulus; Dif: difference (CS+ minus CS-); IBI: inter-beat-interval; VAS: visual analogue scale.

Acoustic startle responses

Line graphs showing changes in mean startle magnitude (elicited during CS presentation) across the post-acquisition and extinction phases are given in Figure S2, separately for each experimental condition:

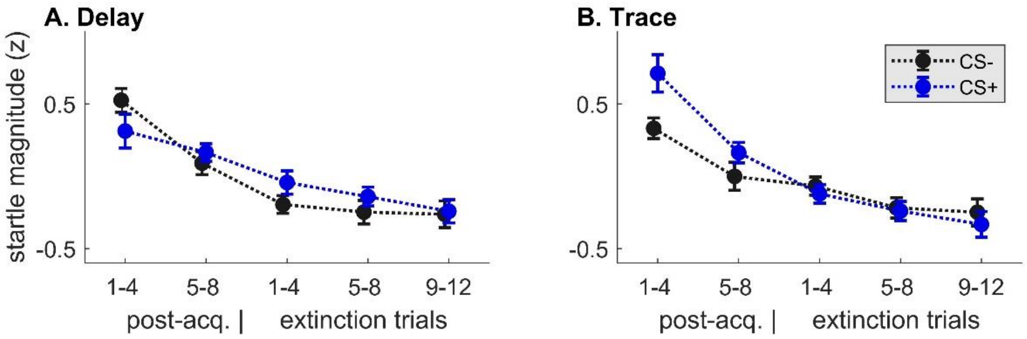

Figure S2. Trajectory of acoustic startle responses elicited during CS presentation (z-scores) across post-acquisition and extinction trials in A. delay and B. trace conditioning.

Note. Error bars: *M*±*SEM*. CS+: conditioned stimulus paired with monetary reward; CS-: never reinforced control stimulus. Graph includes data from all 57 participants with valid startle data.

References

Blumenthal TD, Cuthbert BN, Filion DL, Hackley S, Lipp OV, van Boxtel A. Committee report: Guidelines for human startle eyeblink electromyographic studies. *Psychophysiology* 2005; 42(1):1–15.
